# Study on the Corrosion Behavior of Low-Carbon 9Cr-ODS Steel in Oxygen-Saturated Lead–Bismuth Eutectic for 1000 Hours

**DOI:** 10.3390/nano15040258

**Published:** 2025-02-08

**Authors:** Chongdou Yang, Tao Liu, Yiqun Yang, Youqi Wang, Yuwen Xu, Di Yun, Penghui Lei, Jie Qiu

**Affiliations:** 1 School of Nuclear Science and Technology, Xi’an Jiaotong University, Xi’an 710049, China; 425364@stu.xjtu.edu.cn (C.Y.);; 2 College of Food and Bioengineering, Qiqihar University, Qiqihar 161006, China; 3 Department of Nuclear and Radiological Engineering, University of Illinois Urbana-Champaign, Plasma, Urbana, IL 61801, USA; 4 State Key Laboratory of Multiphase Flow, Xi’an Jiaotong University, Xi’an 710049, China

**Keywords:** corrosion, 9Cr-ODS steel, lead–bismuth eutectic, inner oxide zone

## Abstract

A novel low-carbon 9Cr-ODS steel was exposed to corrosion in lead–bismuth eutectic saturated with oxygen at 500 °C for 1000 h, leading to the formation of three distinct layers of oxide film. From the outermost to the innermost layer, these included a Fe_3_O_4_ layer infiltrated with Pb, a FeCr_2_O_4_ layer, and an inner oxide zone. The inner oxide zone was primarily composed of an unoxidized matrix and Cr_2_O_3_. The formation of the inner oxide zone was primarily attributed to the preferential oxidation of Cr following the infiltration of insufficient O content. Two distinct morphologies of the inner oxide zone were identified: one is porous, while the other is non-porous. The porous morphology is characterized by low Fe content and Pb infiltration. The loss of Fe is the main factor contributing to the development of the porous inner oxide zone and the infiltration of Pb, while the short-range diffusion of Cr promotes the growth of Cr_2_O_3_, resulting in a needle-like morphology.

## 1. Introduction

Energy is a fundamental issue confronting human development. As a form of clean energy, nuclear energy is considered to be one of the key potential solutions to address the energy crisis [1]. Lead-cooled fast reactors (LFRs), a type of fourth-generation nuclear reactor, have garnered significant attention [2]. This interest is primarily attributable to the use of Pb or lead–bismuth eutectic (LBE) as a coolant. The excellent neutron properties of LBE enable an increase in the reactor’s energy density, facilitating its miniaturization and modular design. Furthermore, Pb or LBE could also be used as heavy metal targets for the Accelerator Driven Subcritical System (ADS), which represents an advanced technology for the treatment of nuclear waste within the current nuclear energy sector [3]. Pb and LBE are extensively utilized in nuclear science; thus, issues associated with LBE have been a prominent topic of research in recent years [4]. However, LBE exhibits high solubility with metallic elements, making it highly corrosive to structural materials [5,6]. The structural materials utilized in an LBE environment must withstand a harsh combination of long-term high temperatures, significant radiation damage, strong corrosion, and mechanical stress, all of which pose substantial challenges to the long-term stable operation of the reactor.

Oxide Dispersion Strengthened (ODS) steel is a type of steel known for its excellent radiation resistance [7,8,9,10]. It mitigates radiation-induced defects in the material by dispersing nano-scale Y_2_O_3_ particles throughout the steel. Recently, the Institute of Metal Research at the Chinese Academy of Sciences developed a novel low-carbon 9Cr-ODS steel [11,12]. This new material circumvents the formation of the M_23_C_6_ phase—a precipitated phase that tends to grow at elevated temperatures and adversely affects the material’s properties—by reducing the carbon content [13,14,15,16]. V and N elements are added to form VN precipitates to compensate for the loss of material strength in reducing M_23_C_6_. After a 3000 h, 700 °C high-temperature test, it was found that the comprehensive mechanical properties and microstructure of low-carbon 9Cr-ODS steel remained stable [12]. Xu et al. [17] used 3Mev Fe ion to conduct an experiment at 550 °C under the condition that the irradiation damage reached 70 dpa, and found that the micro-precipitated phase remained stable as a whole. Therefore, low-carbon 9Cr-ODS steel is expected to solve the problem of irradiation and high temperature at the same time. However, its corrosion performance requires further in-depth study.

Currently, one of the primary solutions to the issue of LBE corrosion involves the formation of a protective oxide film [18,19,20]. It is generally observed that when the oxygen partial pressure exceeds 10–6 wt.%, steel containing Fe and Cr will develop a double-layer oxide film [21,22,23]. The inner oxide layer (IOL) typically consists of dense, protective FeCr_2_O_4_, while the outer oxide layer (OOL) is usually Fe_3_O_4_. Du et al. [24] investigated the corrosion of 9Cr-ODS steel after heat treatment in oxygen-saturated LBE at 600 °C for 500 h, and found that a double-layer oxide film was formed, with the inner oxide layer providing effective protection and showing no infiltration of LBE. Zhu et al. [25] investigated the corrosion of ODS-FeCrAl steel in oxygen-saturated LBE at 550 °C for over 8000 h. They found that a double-layer oxide film was formed, with the IOL playing a significant protective role. In general, IOLs are crucial for enhancing corrosion resistance. Currently, the predominant theory regarding the growth mechanism of the double-layer oxide film formed during LBE corrosion is the available space model [26,27,28]. This model posits that Fe is the primary factor driving the growth of the oxide film. The expansion of the outer Fe_3_O_4_ oxide layer results from the outward diffusion of Fe, while the formation of FeCr_2_O_4_ in the IOL is attributed to vacancies created by Fe diffusion, which facilitates the inward diffusion of O. However, some experiments have demonstrated that, during the LBE corrosion process, a chromium-rich region, referred to as the internal oxide zone (IOZ), often forms beneath the IOL [29,30,31]. Zhao et al. [32] corroded 9Cr ferritic–martensitic steel in LBE with an oxygen content of 10–6 wt.% at 550 °C for 1750 h, and they discovered the presence of an IOZ in addition to the double-layer oxide film. Yeliseyeva et al. [33] conducted corrosion tests on 316L steel using oxygen-saturated LBE at temperatures exceeding 500 °C for a duration of 1000 h, which formed an obvious IOZ below the oxide film. The formation of an IOZ is commonly observed in long-term corrosion experiments [34]. This phenomenon can be attributed to the fact that, with sufficient corrosion time, the IOL will thicken, effectively reducing the rate of oxygen diffusion into the matrix. At this stage, from a thermodynamic perspective, the O content at the interface between the oxide film and the matrix (O/M) is insufficient to oxidize the iron in the matrix to form FeCr_2_O_4_. Instead, it leads to the formation of Cr_2_O_3_, which contributes to the development of an IOZ. In this study, we identified two distinct morphologies of IOZs: porous and non-porous, with a particular interest in the former. According to the available space model, the IOZ region is expected to gradually transition into an IOL. The presence of a porous IOZ may ultimately lead to a reduction in the density of the IOL or even to the detachment of the oxide film, thereby compromising the material’s corrosion resistance. Additionally, from the perspective of elemental uniformity, this may result in localized depletion of chromium, adversely affecting the uniformity of the matrix properties. Therefore, we contend that the impact of IOZs on corrosion performance should not be overlooked. In the literature reviewed by the authors, the porous morphology of IOZs resulting from LBE corrosion has not been previously studied; therefore, it is essential to advance the investigation of IOZs further.

In this study, we investigated the corrosion of the oxide film on low-carbon 9Cr-ODS steel in oxygen-saturated LBE at 500 °C for 1000 h. Our findings revealed the presence of an IOZ below the IOL, which was categorized into porous and non-porous morphologies. Both morphologies of the IOZ were meticulously characterized using scanning electron microscopy (SEM) and transmission electron microscopy (TEM), and the mechanisms underlying IOZ formation were elucidated. This research aims to enhance our understanding of the corrosion mechanisms affecting steel in LBE environments.

## 2. Materials and Methods

### 2.1. Materials

The low-carbon 9Cr-ODS steel utilized in this experiment was supplied by the Institute of Metal Research, Chinese Academy of Sciences, with the specific production process detailed in previous work [17]. Upon receipt, the sample weighed approximately 1 kg and was in the form of a cuboid block. After being polished to a smooth finish with 400 mesh SiC sandpaper, the dimensions of the cuboid sample were reduced to approximately 3 × 2 × 0.2 cm via wire cutting. Subsequently, all six surfaces were polished to a glossy finish using 400, 600, 1000, and 2000 mesh SiC sandpaper, followed by 1 µm and 0.5 µm alumina polishing liquids. The chemical composition of the matrix elements is presented in Table 1.

### 2.2. Corrosion Test

The corrosion tests were conducted in a tubular furnace under an argon atmosphere with a flow rate of approximately 50 mL/min. The composition of the LBE consisted of 44.5 wt.% Pb and 55.5 wt.% Bi. During the preparation process, 0.013 wt.% PbO and 0.016 wt.% Bi_2_O_3_ were added to inhibit the reaction between dissolved oxygen and LBE [17]. Prior to the corrosion test, 400 g of solid LBE was placed in an alumina crucible and heated to 200 °C in a muffle furnace to facilitate melting. The sample was secured on 316L steel wire and subsequently introduced into the crucible. The crucible was then positioned in a tubular furnace, and the LBE was heated to 500 °C for the corrosion experiments. At the conclusion of the experiment, solid yellow oxides (PbO and Bi_2_O_3_) were observed floating on the surface of the crucible, while the crucible’s interior contained molten LBE. This phenomenon can be attributed to the lower density and higher melting points of PbO (888 °C) and Bi_2_O_3_ (860 °C) when compared to LBE. The formation of PbO and Bi_2_O_3_ at 500 °C necessitates high oxygen concentrations of 10^−4^ wt.% and 10^−3^ wt.%, respectively, which exceed the oxygen saturation level of LBE (approximately 10^−3^ wt.%). Thus, it can be inferred that the experiment achieved oxygen-saturated conditions. After the corrosion test, the sample was removed and allowed to cool to room temperature. The sample was then cold-set using epoxy resin and coagulant. The corroded section was polished using 400, 600, 1000, and 2000 mesh SiC sandpaper, followed by 1 µm and 0.5 µm alumina polishing liquid, until a smooth, mirror-like finish was achieved.

### 2.3. Microstructure Characterization

After the corrosion experiments, we employed SEM and TEM to analyze the oxide film resulting from corrosion. A gold coating was applied to the cross-section of the sample to enhance its electrical conductivity. MC1000 gold spraying equipment from Hitachi, Tokyo, Japan, was utilized, which includes the MC1000 main unit, an HHTNT-CT-2000 transformer, a gold target material, and a Hitachi vacuum pump. Subsequently, microstructural and elemental analyses were conducted using a scanning electron microscope (SEM, Gemini 500, ZEISS Sigma, Cambridge, UK) and an energy dispersive spectrometer (EDS). The TEM specimen was prepared by the SEM microscope equipped with a Ga-focused ion beam (FIB, 30 kV, Thermo Scientific Scios 2, Thermo Fisher Scientific, Waltham, MA, USA) and an Omniprobe manipulator. The pieces were initially pre-cut from the bulk samples by using a current of 7 nA, and then ion beam currents from 0.5, 0.3, and 0.1 nA to 10 pA were used in sequence to further mill the piece into electron-transparent slices with a thickness of 70 nm [24]. The microstructure of the samples was characterized by TEM (Talos 200 kv, Thermo Fisher Scientific, Waltham, MA, USA and JL-F200, JEOL, Tokyo, Japan).

## 3. Results

**Figure 1:**
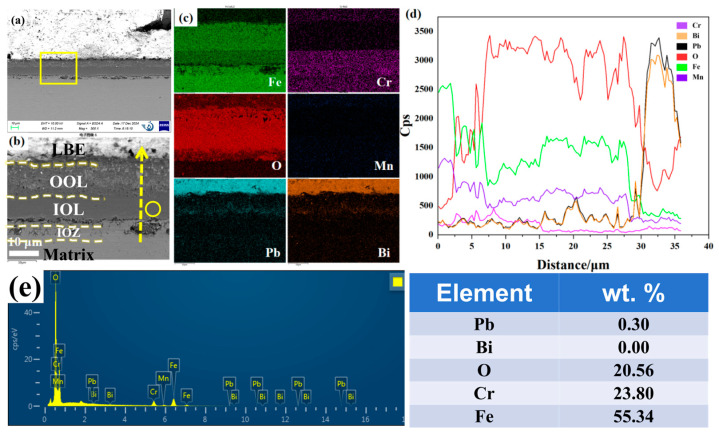
Cross-sectional SEM results of the sample after 1000 h of corrosion at 500 °C: (**a**) morphology in backscattered electron (BSE) mode; (**b**) magnification corresponding to the yellow box in (**a**); (**c**) EDS mappings of Fe, Cr, Mn, O, Pb, and Bi corresponding to (**b**); (**d**) EDS line scan of Fe, Cr, Mn, O, Pb, and Bi along the yellow arrow in (**b**); (**e**) Point energy spectrum analysis corresponding to the yellow circle position in Figure 2a.

Figure 1 shows the cross-sectional morphology of the oxide film and the EDS results obtained via SEM following corrosion. The image was captured using the BSE mode, where the brighter areas correspond to regions rich in heavy elements, particularly LBE. As depicted in Figure 1a, a uniform oxide film approximately 25 μm thick formed on the substrate surface after corrosion. The magnified view of the yellow region is presented in Figure 1b. Integrating the EDS results from Figure 1c,d, we observe that the oxide film is stratified into three distinct layers. The OOL appears porous, exhibiting numerous gray spots, and energy spectrum analysis indicates it is infiltrated with LBE and primarily composed of Fe and O. The inner oxide layer (IOL) demonstrates robust overall protection, with energy spectrum results showing it mainly contains Cr, Mn, O, and Fe, while fluctuations in the Pb peak within the IOL are also noted. Notably, we identified an inner oxidation zone (IOZ) rich in Cr and O located beneath the IOL. The IOZ exhibits two predominant morphologies: one is porous, and the other is non-porous. Figure 1d reveals that the IOZ region is enriched with Cr, Mn, and O but poor in Fe, with a corresponding increase in Pb. We are particularly interested in the morphology variations within the IOZ region.

**Figure 2:**
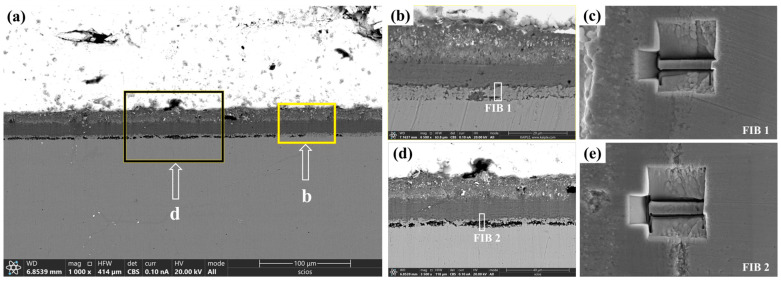
Cross section of the oxide film observed under a SEM using a focused ion beam. (**a**) shows the topography in BSE mode; (**b**,**d**) correspond to the magnified images in (**a**), while (**c**,**e**) indicate the specific locations of the FIB cutouts, observed in SE2 mode.

To further investigate the IOZ region, we selected both porous and non-porous IOZ samples, which were prepared using a focused ion beam (FIB). Figure 2 illustrates the specific locations from which the FIB samples were extracted. As shown in Figure 2a, under BSE-SEM observation, the IOZ exhibits two distinct morphologies: one is porous, represented by the deep black region, and the other is non-porous. We prepared FIB samples from IOZs with different morphologies, as depicted in Figure 2b,d. Figure 2c,e displays the morphologies observed in secondary electron mode (SE2) by SEM during the extraction of the FIB samples. It is evident that FIB1 originates from the non-porous area of the IOZ, while FIB2 is derived from the porous area.

**Figure 3:**
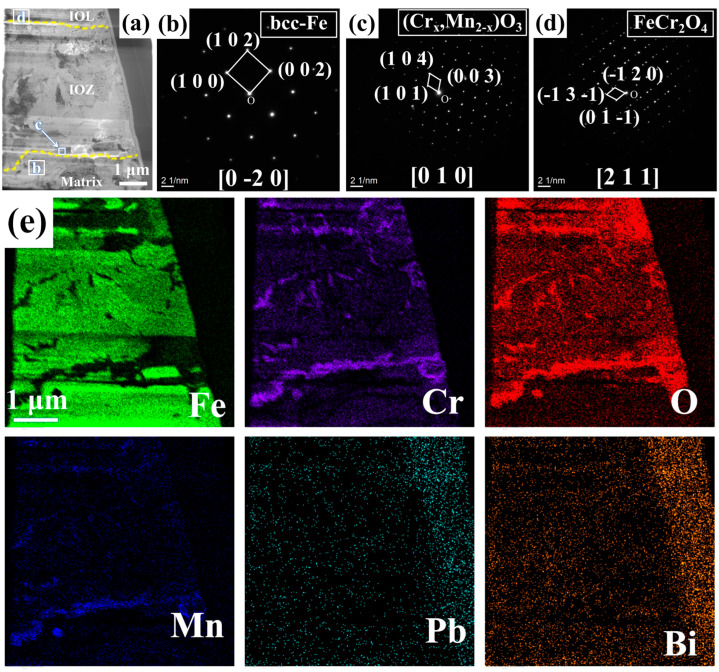
FIB1 images observed under TEM: (**a**) morphology in BF mode; (**b**–**d**) correspond to SAED pattern images from different positions marked in (**a**); (**e**) displays the EDS mappings of Fe, Cr, O, Mn, Pb, and Bi.

Figure 3 presents the BF model morphology, SAED pattern images, and EDS mappings of FIB1 under TEM observation. As shown in Figure 3a, even the FIB1 sample extracted from the non-porous area contains a small number of pores, which is also reflected in the morphology depicted in Figure 2c. SAED analysis was conducted at various locations on the sample, as illustrated in Figure 3b–d. Combining these results with the element mappings in Figure 3e, we can identify Fe with a bcc structure at location b (PDF#50-1275), Cr_2_O_3_ with Mn at location c (PDF#38-1479), and FeCr_2_O_4_ at location d (PDF#24-0511). Notably, Cr_2_O_3_ forms a coherent layer approximately 0.3 μm thick along the junction of the IOZ and the matrix, with negligible O element present beneath this layer, while there is a partial enrichment of O and Cr on it. Given that the signals for Pb and Bi are weak and the rightmost position corresponds to noise during energy spectrum acquisition, this indicates that there is essentially no Pb infiltration in the non-porous IOZ region.

**Figure 4:**
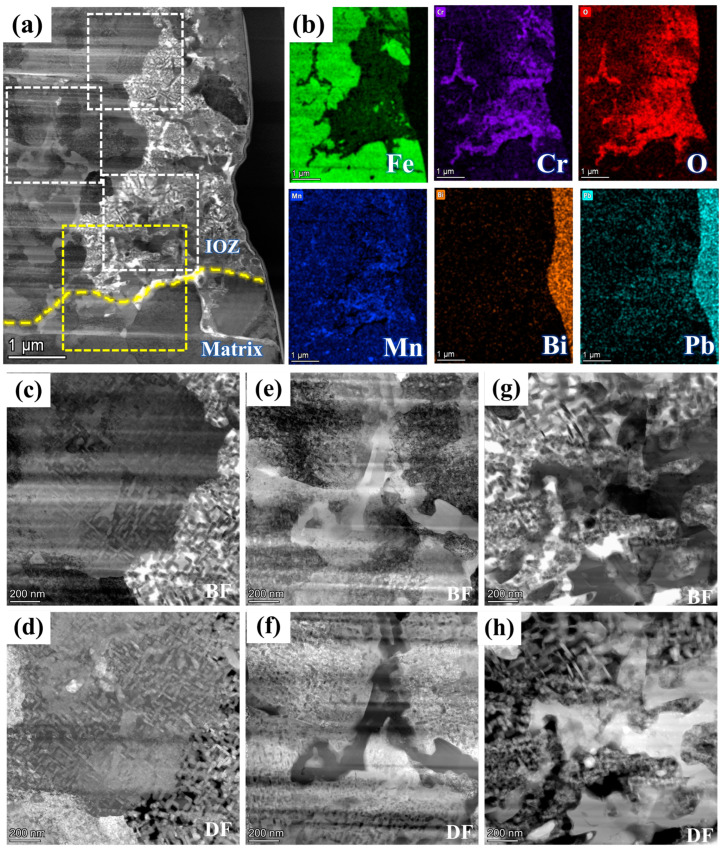
FIB2 images observed under TEM: (**a**) morphology in HAADF mode; (**b**) shows the EDS mappings of Fe, Cr, O, Mn, Pb, and Bi; (**c**,**e**,**g**) and (**d**,**f**,**h**) are bright and dark field images corresponding to different positions in (**a**), respectively. The white dotted box area from top to bottom corresponds to (**c**,**d**), (**e**,**f**), (**g**,**h**) respectively. The yellow dotted box corresponds to Figure 5.

**Figure 5:**
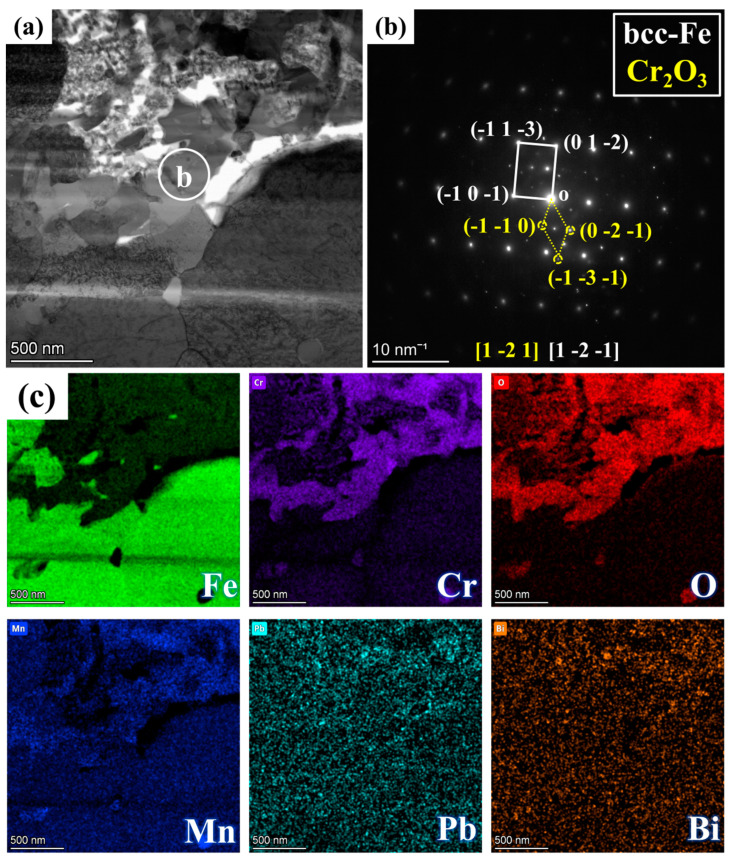
Magnified image of the yellow box area in Figure 4a: (**a**) BF image, (**b**) SAED pattern corresponding to region b in (**a**), and (**c**) mappings of Fe, Cr, O, Mn, Pb, and Bi.

Figure 4 presents the BF model morphology and EDS mappings of FIB2 under TEM observation. Figure 2b illustrates that the porous regions contain minimal Fe elements, yet are abundant in Cr, Mn, and O elements, along with some Pb infiltration. Additionally, a region rich in Cr, Mn, and O is observed at the junction of the matrix and the IOZ, which aligns with the findings presented in Figure 3e. The further amplification of various regions depicted in panel a is shown in Figure 4c–h, utilizing BF and DF image comparisons to assess the presence of precipitated phases. Numerous acicular precipitates are identified within both the matrix and porous regions, along with many fine grains in the porous area. A more detailed examination of the O/M junction, as illustrated in Figure 5, corresponds to the yellow box area in Figure 4a, revealing the presence of numerous pores and fine grains at the junction. Additionally, SAED analysis was conducted on region b, with the results presented in Figure 5b. The calibration results, combined with the energy spectrum data in Figure 5c, indicate that the phase present is Cr_2_O_3_ and bcc-Fe. It is evident that the Fe content in the porous region is low, primarily enriched with Cr, Mn, and O, along with evidence of Pb and Bi infiltration.

**Figure 6:**
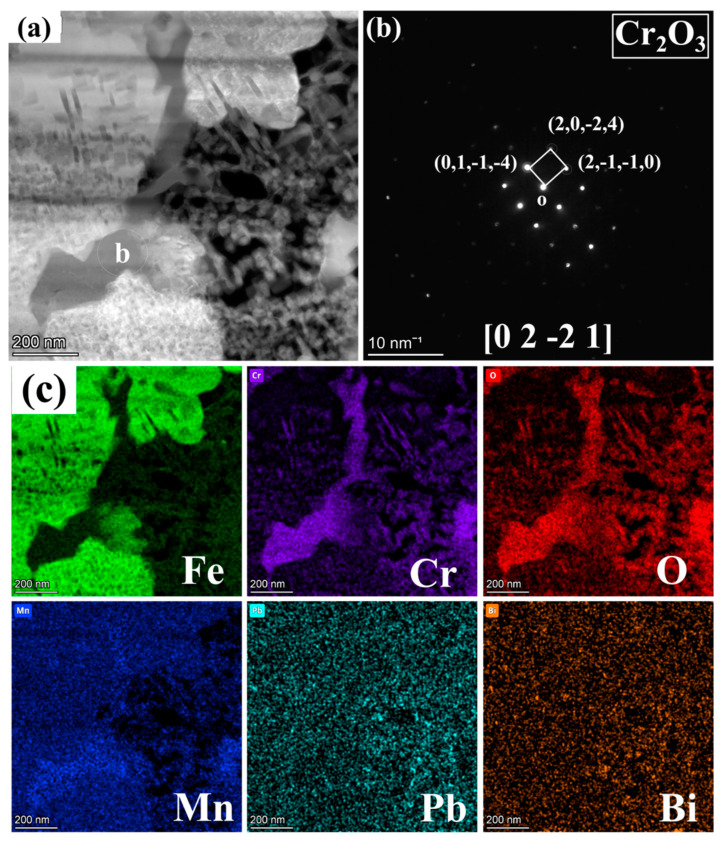
Magnified image of Figure 4e: (**a**) BF image, (**b**) SAED pattern corresponding to region b in (**a**), and (**c**) mappings of Fe, Cr, O, Mn, Pb, and Bi.

**Figure 7:**
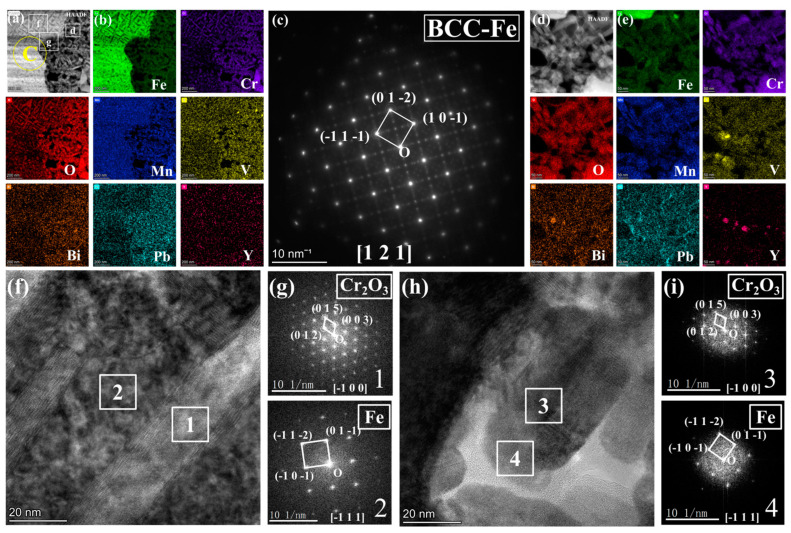
(**a**) corresponds to the magnified HAADF image shown in Figure 4c; (**b**) EDS mappings of Fe, O, Mn, Cr, and Pb for (**a**); (**c**) presents the SAED pattern image for region b of a; (**d**) illustrates the enlarged HAADF image of region d of a; (**e**) EDS mappings of Fe, O, Mn, Cr, and Pb for (**d**); (**f**) includes the HRTEM image corresponding to region f in a; (**g**) provides the FFT pattern images for regions 1 and 2 in f; (**h**) displays the HRTEM image corresponding to region g in a; (**i**) presents the FFT pattern images for regions 3 and 4 in h.

Figure 6 further illustrates the morphology, SAED analysis, and EDS mapping analysis of different elements in the region of Figure 4e under magnified HAADF mode. At the boundary between the porous and non-porous regions, a zone rich in light elements is observed, with the grains identified as Cr_2_O_3_ through SAED analysis, corroborated by the energy spectrum results in Figure 6c. Notably, in these acicular oxides, the Cr depletion zone alternates with the Fe depletion zone. Generally, areas rich in Cr oxides exhibit very low Fe content, while regions containing Fe show significantly reduced Cr levels. Mn is present between these two regions. To further investigate this phenomenon, the non-porous and porous regions of the IOZ are analyzed in Figure 7. The results in Figure 7a,b clearly indicate substantial Pb infiltration near these voids. Given the absence of hole positions in this area, it can be concluded that this is not due to noise interference. Moreover, the presence of Pb is consistent with the line scan results in Figure 1d. Figure 7d further magnifies the porous IOZ region, revealing that Pb infiltration primarily occurs near the voids. Additional HRTEM analysis of the needle-like region shows that Fe and Cr_2_O_3_ appear alternately throughout all non-porous IOZ regions, with numerous voids present. In porous regions, Fe and Cr_2_O_3_ also exhibit an alternating pattern. Specifically, Figure 7g,i demonstrates a clear orientation relationship between the areas of Cr_2_O_3_ and Fe ([1-21] Fe//[1-2-1]Cr_2_O_3_), indicating a close correlation between the oxidation of Cr and the matrix.

## 4. Discussion

### 4.1. Oxidation Mechanism of the OOL and IOL

After 1000 h of exposure to low-carbon 9Cr-ODS steel in LBE saturated with oxygen at 500 °C, three layers of oxide film were formed: the outer layer, the inner layer, and the IOZ layer (see Figure 1, Figure 3 and Figure 4). The inner layer is a relatively dense FeCr_2_O_4_, while the outer layer is Fe_3_O_4_ that has been infiltrated by LBE. The characteristics of the IOZ layer are discussed in Section 4.2. In our previous experiments, corrosion tests were conducted on steel samples for durations of 1 h, 100 h, and 200 h under identical conditions [35]. The results indicated that the IOL consisted of FeCr_2_O_4_, while the outer layer was identified as Fe_3_O_4_. Therefore, considering that the EDS results presented in Figure 1c are consistent with previous findings, it is reasonable to conclude that the outer layer is indeed Fe_3_O_4_.

**Figure 8:**
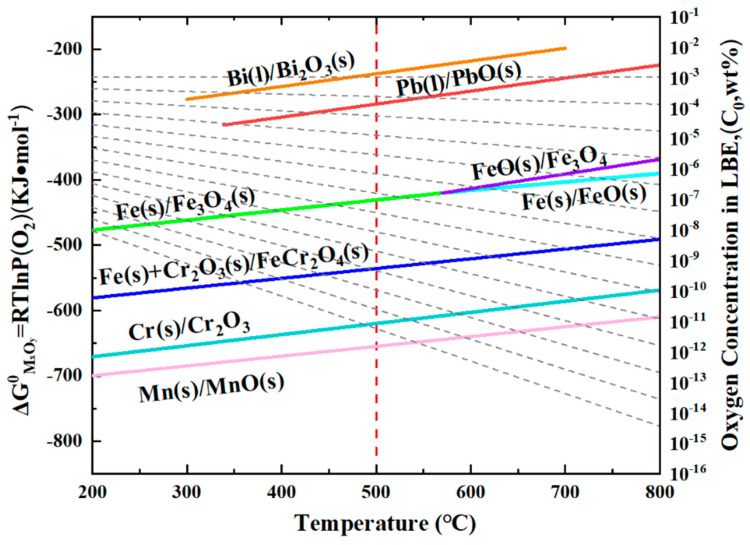
Ellingham diagrams of the standard Gibbs free energy of the concentration of dissolved oxygen in liquid LBE [37].

The mechanism underlying the formation of a double-layer oxide film during lead–bismuth corrosion can be elucidated using a widely accepted usable space model [26,27,28]. This model posits that the diffusion of Fe is critical to the growth of the oxide film. Figure 8 illustrates the thermodynamic Ellingham diagram, which indicates that for different oxides, the lower the Gibbs free energy, and the more readily they tend to form preferentially [36]. As Fe diffuses outward, it reacts with O in LBE, leading to the deposition of an Fe_3_O_4_ layer. Concurrently, as Fe moves outward and creates vacant sites, O infiltrates the matrix and reacts with Cr to form Cr_2_O3. As illustrated in Figure 8, when the O content is adequate, Fe will further react with Cr_2_O_3_ to produce FeCr_2_O_4_. The oxygen concentration in molten LBE can be calculated using the following formula [6,36]:

logC=A−B/T

where C represents the mass percentage concentration of dissolved oxygen, A and B are constants, and T denotes the temperature in Kelvin. According to the literature, at approximately 500 °C, the concentration of dissolved oxygen in LBE ranges from 10^−2^ to 10^−3^ wt.%. This level of oxygen is sufficient for Cr_2_O_3_ to react with Fe, resulting in the formation of FeCr_2_O_4_. Therefore, the substrate surface will gradually develop a double-layer oxide film. The relatively dense nature of FeCr_2_O_4_ in the inner layer, which provides effective protection, can be attributed to two factors. First, FeCr_2_O_4_ becomes denser upon expansion due to its high Pb ratio [37,38,39]. Second, in the spinel structure, Cr^3+^ occupies the tetrahedral sites, while Fe^2+^ occupies the octahedral sites. Consequently, Fe has limited diffusion through the tetrahedral gaps but can diffuse through the octahedral gaps [26,27,28]. This mechanism effectively reduces the diffusion rate of Fe, thereby slowing down the corrosion rate. In contrast, the outer layer is directly exposed to LBE, leading to the dissolution of Fe into LBE and the formation of a porous Fe_3_O_4_ structure. This allows LBE to penetrate the vacancies created by the loss of Fe, resulting in the observed phenomenon of LBE ingress into the outer layer, as illustrated in Figure 1c. Moreover, it is evident that the infiltration of Pb is significantly more pronounced than that of Bi, as demonstrated by numerous studies. One possible explanation is that Pb absorption is stronger than that of Bi, making it easier to penetrate [36].

### 4.2. Oxidation Mechanism of IOZ

The IOZ primarily consists of Cr_2_O_3_ and an unoxidized Fe matrix. In many regions, the oxidized Cr exhibits a needle-like morphology, while relatively coherent Cr_2_O_3_ is present in the OM (see Figure 3, Figure 4 and Figure 5). The following discussion explores the relationship between the IOZ and corrosion time as well as the factors contributing to the formation of porous and non-porous IOZs.

We conducted corrosion tests on this steel for 1 h, 100 h, and 200 h, and no significant IOZ formation was observed. However, when the corrosion time was extended to 1000 h in this experiment, IOZ formation was detected. This occurs because, with prolonged corrosion time, the oxide film thickness increases, enhancing the protective layer, which slows down oxygen infiltration and leads to the preferential oxidation of Cr. In summary, the protection provided by the IOL is the primary reason for the formation of the IOZ. Cr_2_O_3_ exhibits the lowest Gibbs free energy, indicating that Cr has the highest oxidation priority [36]. When the oxygen content is sufficient, Cr_2_O_3_ can continue to react with Fe to form FeCr_2_O_4_. However, as the IOL becomes progressively thicker, the oxygen concentration at the interface between the matrix and the IOL decreases. Consequently, Cr can only be preferentially oxidized under these conditions. Due to the grain boundary serving as a fast diffusion channel, oxygen preferentially penetrates along the grain boundaries and oxidizes them, which accounts for the tree-like morphology observed in the IOZ. As O approaches the depleted position, Cr and Mn are preferentially oxidized, resulting in the formation of a coherent layer of Cr_2_O_3_ interspersed with Mn (see Figure 3, Figure 4 and Figure 5). Chen et al. [40] investigated the corrosion of T91 steel in supercritical water for 1500 h and identified four distinct oxidation types. The outer layer was composed of Fe_3_O_4_, while the inner layer consisted of FeCr_2_O_4_. The primary component of the IOZ was preferentially oxidized chromium, and coherent Cr_2_O_3_ was also detected at the outer margin. The authors propose that when the Cr/O ratio reaches a certain critical threshold, Cr_2_O_3_ is generated concurrently with the depletion at the edge, which aligns with the observations from our experiment.

There are two primary morphologies of the IOZ: non-porous and porous. As shown in Figure 2c,e, the SE2 mode of TEM reveals that the observed IOZ already exhibits distinct porous regions. Therefore, these voids are not a result of FIB excisions but are pre-existing. Both the porous and non-porous IOZs contain acicular Cr_2_O_3_ (see Figure 7). The most significant difference is that the porous IOZ is markedly Fe-deficient (Figure 3 and Figure 4). For these needle-like Cr_2_O_3_ structures, the uneven distribution of Cr within the matrix can be ruled out based on previous characterizations [17]. Therefore, the formation of acicular oxides is likely due to the selective oxidation of O after it infiltrates the matrix. With the increase of O infiltration and Cr diffusion in the short range, the Cr-rich oxide will grow and appear with a needle-like morphology, and create a Cr-poor zone around it. Ye et al. [41] investigated the corrosion of T91 steel in oxygen-saturated liquid LBE at 550 °C for 2000 h. They observed that the chromium oxides in the IOZ exhibited a needle-like distribution, with chromium-poor regions forming around them. Similarly, Zhang et al. [42] found that after SIMP steel was corroded in oxygen-saturated LBE at 600 °C for 2000 h, needle-like Cr_2_O_3_ and FeCr_2_O_4_ phases emerged in the IOZ, demonstrating a significant orientation relationship with the matrix. The formation of this acicular Cr_2_O_3_ is attributed to the short-range diffusion of Cr in the matrix and its oxidation growth at grain boundaries or within the crystal. As mentioned earlier, O infiltration preferentially oxidizes along grain boundaries. However, the primary grain boundaries are mainly VN. Although previous characterizations showed a few precipitates coupled with VN and Cr, most grain boundaries do not contain Cr. This implies that, in addition to the diffusion of Cr to the grain boundaries, O will gradually infiltrate the grain interior, leading to internal oxidation and the formation of Cr-depleted regions. These regions create a concentration gradient, facilitating the diffusion of surrounding Cr to these depleted areas. The nucleation and growth of Cr_2_O_3_ then result in the needle-like structure observed. Figure 7g,i illustrates a clear orientation relationship between Cr_2_O_3_ and Fe areas, indicating that Cr oxides initially form in the matrix and subsequently grow into a needle-like morphology (Figure 6 and Figure 7).

The diffusion of Fe primarily depends on the concentration gradient of Fe and the concentration of O [43,44,45]. The presence of O promotes the outward diffusion and oxidation of Fe. The Fe-deficient phenomenon in the porous IOZ suggests significant Fe diffusion. In some areas, a minor amount of LBE might have infiltrated the IOL, likely due to prolonged corrosion time. In Figure 1d, Pb peaks are observed in both the IOL and IOZ regions, and point scan results corroborate Pb infiltration in these areas. The slow infiltration of Pb can serve as a nanochannel for Fe and O diffusion, thereby increasing the Fe diffusion rate. Figure 4, Figure 5, Figure 6 and Figure 7 show a low Fe content in the porous regions, supporting our hypothesis. Additionally, O can enter the IOZ via infiltrated Pb and extensively oxidize the residual Cr, explaining the high Cr oxidation near the porous IOZ (see Figure 4). Some studies have proposed that an IOZ will gradually transform into an IOL as the corrosion process progresses [4,34]. Therefore, over time, the porous IOZ may affect the densification of a subsequently grown IOL, negatively impacting the material’s long-term corrosion resistance.

## 5. Conclusions

In this study, we corroded low-carbon 9Cr-ODS steel in oxygen-saturated liquid LBE at 500 °C for 1000 h. SEM and TEM were employed to characterize the cross section of the oxide film that formed after corrosion in detail. The conclusions are as follows:

1. After 1000 h of corrosion at 500 °C, three distinct layers of oxide film were formed on the substrate’s surface: an Fe_3_O_4_ layer infiltrating the LBE, a dense FeCr_2_O_4_ layer, and an IOZ layer arranged from outside to inside.
2. The formation of the Fe_3_O_4_ and FeCr_2_O_4_ layers is attributed to the diffusion of Fe. The outward diffusion of Fe leads to the growth of the Fe_3_O_4_ layer, while the inward diffusion of O results in the formation of the FeCr_2_O_4_ layer.
3. The IOZ primarily consists of unoxidized matrix and Cr_2_O_3_, exhibiting two morphologies: one porous, and the other non-porous.
4. The predominant oxide in the IOZ is Cr_2_O_3_. This is attributed to the dense oxide film, which reduces the O content near the substrate and preferentially facilitates the formation of Gibbs free energy associated with low Cr_2_O_3_.

## Figures and Tables

**Table 1:** Nominal composition of 9Cr-ODS steel used in this study (wt.%).

Fe	Cr	Mn	N	W	Ta	V	Y	O	C	Si
Bal.	8.82	0.96	0.12	0.99	0.097	0.39	0.21	0.16	0.011	0.009

## Data Availability

The raw data required to reproduce these findings cannot be shared at this time as the data also forms part of an ongoing study.
